# The focal index: a quantitative approach to morphological sub-phenotyping of COVID-19 patients with acute respiratory distress syndrome: a pilot study

**DOI:** 10.1186/s40635-025-00794-0

**Published:** 2025-08-08

**Authors:** Kristin Jona Bjarnadottir, Martin Tovedal, Gaetano Perchiazzi, Miklos Lipcsey, Lucian Covaciu, Magnus von Seth, Rafael Kawati, Mariangela Pellegrini

**Affiliations:** 1 Intensive Care Unit, Department of Anaesthesia, Perioperative Medicine and Intensive Care, Uppsala University Hospital, Uppsala, Sweden; 2 Hedenstierna Laboratory, Department of Surgical Sciences, Uppsala University, Akademiska Sjukhuset, Ing 40, 3 tr, 751 85 Uppsala, Sweden

**Keywords:** Acute respiratory distress syndrome, Lung imaging, Sub-phenotypes, Mechanical ventilation

## Abstract

**Background:**

Acute respiratory distress syndrome (ARDS) is characterised by significant morphological heterogeneity. Morphological sub-phenotyping can potentially be used to personalise mechanical ventilation. Current methods to classify lung injury as focal or diffuse rely on subjective image interpretation, which risks misclassification and suboptimal treatment. This study aimed to investigate the morphological appearance features of lung injury objectively. The focal index, an objective quantitative tool, was introduced to assess focality in lung injury.

**Methods:**

In this single-centre retrospective study, we included lung computed tomography (CT) scans from COVID-19 ARDS patients on invasive mechanical ventilation, classified as diffuse lung injury. CT data were analysed to extract regional Hounsfield Unit (HU) profiles across nine predefined lung areas. The focal index was derived by quantifying the non-overlapping area under HU distribution curves between the apical ventral and diaphragmatic dorsal regions. Correlations with lung weight, gas volume, and ventilatory settings were assessed. For validation, at least two experienced ICU consultants assessed the same images and determined whether ARDS was of a diffuse or focal type. The experts classified 36 out of 37 patients as diffuse ARDS, with substantial interobserver agreement (*k* = 0.65, 95% CI 0.02–1.00).

**Results:**

The focal index demonstrated a wide range (25–175; mean 95.5 ± standard deviation 42.8), correlating significantly with the dorsal diaphragmatic non-aerated area (*r* = 0.67, *p* < 0.01) and with total gas volume (*r* = − 0.36, *p* = 0.03). There was no significant influence of ventilatory settings on the focal index.

**Conclusions:**

The analysis suggested diffuse lung injury includes a spectrum of focality rather than a binary classification. The focal index provides an objective method to quantify the focality of lung injury in ARDS. Further studies are needed to validate the focal index across diverse ARDS aetiologies and establish its clinical application threshold for guiding personalised ventilation strategies.

**Supplementary Information:**

The online version contains supplementary material available at 10.1186/s40635-025-00794-0.

## Background

Acute respiratory distress syndrome (ARDS) is a frequent cause of hypoxemic respiratory failure and is characterised by significant heterogeneity [1]. Despite technological advances and the evolution of lung protective ventilation [2], ARDS mortality remains high [3], underlining the limitations of a one-size-fits-all approach for ventilator settings and the need for individualising ventilatory strategies. Identifying sub-phenotypes can help target the treatment of individual patients and improve outcomes [4, 5]. While lung imaging was previously limited to diagnostics [6] and for investigating pathophysiological mechanisms [7–9], its use in morphological sub-phenotyping has received growing interest in ARDS in recent years. The LIVE trial [10] was a randomised clinical study designed to compare conventional clinical practice with personalised ventilator settings based on computed tomography (CT) or chest *X*-ray assessments of lung injury morphology. More recently, lung ultrasound (LUS) [11, 12] has also been investigated as a potential tool to discern between morphological sub-phenotypes of ARDS [12]. The PEGASUS trial is currently studying LUS as a bedside non-invasive alternative to CT for personalising ventilation [13]. For both approaches (i.e., lung CT and LUS), lung injury is categorised as either diffuse or focal, and based on that, two distinct ventilator strategies are applied accordingly. While lungs with predominant dorsal and caudal consolidation (focal injury) benefit more from prone positioning and low positive end-expiratory pressure (PEEP), lungs with diffuse and patchy loss of aeration (diffuse injury) respond better to lung recruitment, PEEP and low tidal volumes [14, 15]. Whereas the PEGASUS trial is ongoing [13], the LIVE study [10], based on lung CT, revealed poor interobserver agreement in classifying patients and an increased mortality risk when patients were misclassified and enrolled into the wrong intervention groups. This underscores the critical yet challenging importance of accurate classification of morphological findings. All proposed methods and algorithms for patient classification based on lung morphology share observer-dependent and subjective assessment of lung images, potentially resulting in conflicting diagnoses [16]. Although tempting and conceptually correct, one can argue that morphological sub-phenotyping should be based on objective scores and thresholds. In this preliminary analysis, we hypothesised that a wide variation of “focality” of the lung injury could be demonstrated even in lung CT scans classified as diffuse. Based on previous evidence of focal and diffuse injury distribution within the lung parenchyma and the corresponding regional profile of the Hounsfield unit [14], we tested a score to quantify the degree of focality for lung CT scans objectively in a cohort of patients with COVID-19-induced ARDS.

## Methods

This single-centre retrospective study was approved by the Swedish National Ethical Review Agency (Dnr 2024–03697-01) and conducted in accordance with the Helsinki Declaration and its most recent amendments. Given the retrospective and anonymous nature of the study, informed consent was not required.

### Patients

The main inclusion criterion was diffuse ARDS, diagnosed by expert consensus using the same criteria as described in the LIVE study [10]. Given the diffuse nature of lung injury during the early phase of COVID-related ARDS, we included patients who underwent either a native no-contrast lung CT or a dual-energy lung CT during their intensive care unit (ICU) stay while on invasive mechanical ventilation. For dual-energy CT, only the virtual non-contrast images were analysed. Only the first CT scan performed after ICU admission was included for patients with more than one CT scan. All the analysed CT scans were performed based on clinical indication. Other inclusion criteria were: (1) age > 18 years; (2) a positive polymerase chain reaction test for SARS-CoV-2 on a nasal swab specimen; (3) acute respiratory failure as the leading cause of ICU admission; (4) diagnosis of ARDS before or contextually to the analysed CT scan. Blinded to each other’s evaluation, the patient’s clinical conditions, and the current analysis aims, three consultants with more than 10 years of ICU experience each evaluated the lung injury’s nature (i.e., diffuse or focal). All images were reviewed by at least two investigators. In case of disagreement between the first two, a third expert was subsequently asked.

### Clinical data

Clinical information, including demographic data, laboratory findings, and respiratory parameters, was collected from patients’ medical records during their ICU care period. Respiratory and ventilator parameters were collected at the time of the CT scan, and laboratory and blood gas analysis results were collected on the same day.

### CT image analysis

**Fig. 1:**
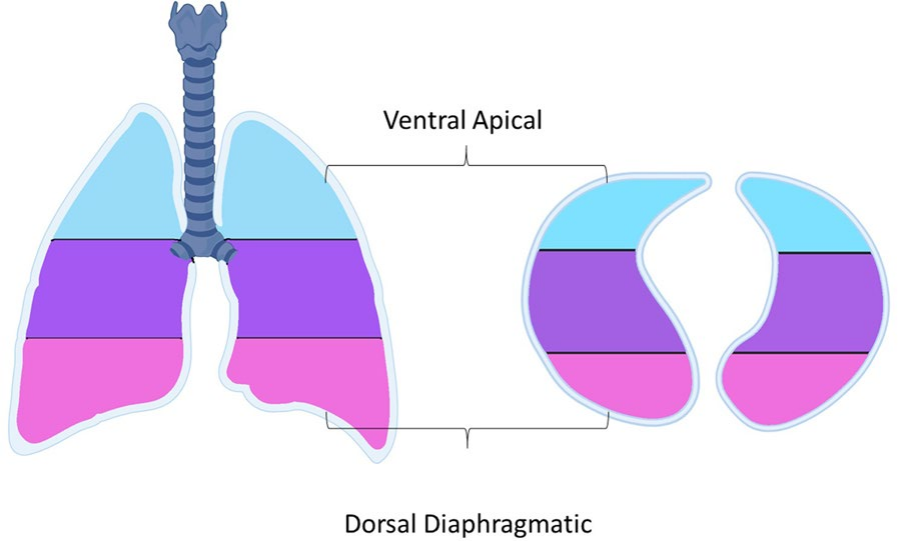
Schematic illustration of the examined lung area divided into nine defined regions. **A**. Anterioposterior division with apical (blue), mediastinal (purple) and diaphragmatic (pink) regions. **B**. Craniocaudal division with ventral (blue), medial (purple) and dorsal (pink) regions

**Fig. 2:**
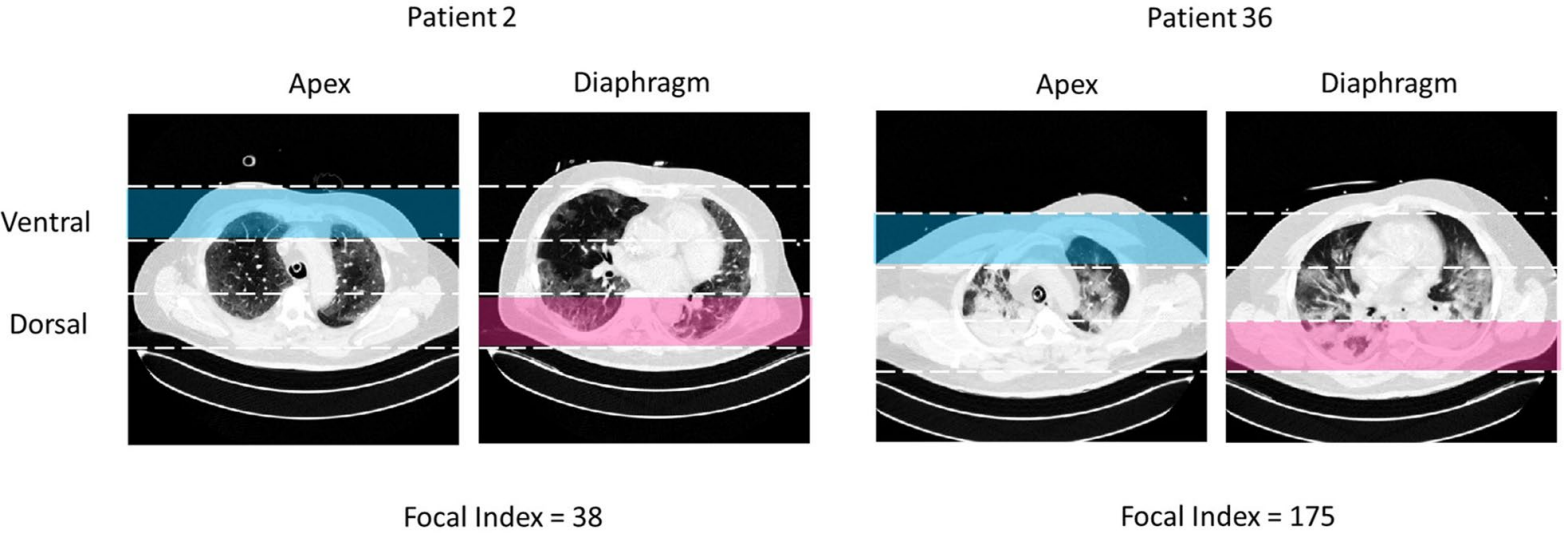
Representative examples of CT scans from the 2 lung regions used to calculate the focal index. Two patients with extreme focal index values are reported. The ventral apical and the dorsal diaphragmatic areas are noted in blue and pink, respectively

CT scans were performed over the whole thorax and in a supine position while the patients were deeply sedated and on controlled mechanical ventilation. For each CT examination, 18 contiguous 1.5-mm-thick images equally distributed along the craniocaudal axis and covering the whole craniocaudal extension of the lungs were selected for the analysis. A semiautomatic image segmentation was applied to extract information from the lung parenchyma. To analyse the Hounsfield unit (HU) distribution profile at a regional level, we divided the selected 18 images into three groups along the craniocaudal axis (i.e., apical, mediastinum, diaphragmatic). After aligning the images along the gravitational axis, three gravitational regions (i.e., ventral, medial, and dorsal) were defined. This way, we obtained nine three-dimensional areas to analyse, and for each, we extracted the HU profile (Figs. 1 and 2). Only voxels corresponding to the lung parenchyma were analysed following lung segmentation, each characterised by a HU value. HU values for the lung (CT lung density) depend on lung aeration and range between − 1000 HU, corresponding to total air, and + 100 HU, corresponding to the non-aerated lung. The HU distribution profile was reported on a Cartesian axis system as a histogram: on the *x*-axis, the HU values, with bin width set at 5 HU, and on the *y*-axis, the percentage of the total voxels reporting a specific HU value. As previously described, the hyperaerated portion of the HU profile was defined by a CT density between − 1000 and − 800 HU, and the non-aerated portion of the HU profile was defined between − 100 and + 100 HU (Additional Figure E1). Weight [g] and total volume of gas [ml] were estimated for each selected scan first [17, 18] and then for the whole lung following the interpolation method [19].

### The focal index

Previous CT studies on ARDS morphology [20] showed that in the case of focal injury, the apical ventral and the diaphragmatic dorsal lung portions are expected to show the highest difference in HU distribution. In contrast, the HU distribution profile for diffuse injury is expected to be comparable among all lung portions. Based on this rationale, the focal index was defined as the non-overlapping area between the HU distribution curves of the ventral apical and dorsal diaphragmatic regions. Letting *f*_VA_(*x*) and *f*_DD_(*x*) denote the respective normalised HU distribution functions of ventral apical and dorsal diaphragmatic regions, and *x* ∈ [− 1000, + 100] the HU range, the focal index was calculated as:

$${\text{Focal Index }} = \, \left( {\int_{x} {\left| {f_{{{\text{VA}}}} \left( x \right) \, - f_{{{\text{DD}}}} \left( x \right)} \right|} dx} \right) \, x \, 100$$

where:

*f*_VA_(*x*) is the HU distribution function of the ventral apical region,

*f*_DD_(*x*) is the HU distribution function of the dorsal diaphragmatic region,

*x* is the HU value ranging from − 1000 to + 100.

Both curves were normalised such that their area under the curve (AUC) equalled 100. Thus, the focal index ranges from 0 (complete overlap between the two regions, indicating homogeneous aeration and no focality) to 200 (no overlap at all, indicating maximal difference and high focality).

The correlations between the focal index and lung weight and between the focal index and overall gas content in the lung were investigated.

### Sensitivity analysis

A multiple linear regression analysis was performed to investigate the possible influence of ventilatory settings (independent variables) on the focal index (dependent variable). The selected variables were: (1) PEEP (1 cmH_2_O), (2) respiratory rate (RR, 1 breath/minute), and tidal volume per predicted body weight (Vt/PBW, 1 ml/kg PBW).

### Statistical analysis

Given the preliminary and descriptive nature of the analysis, no formal sample size calculation was performed. The degree of agreement between the first two observers for categorical data was estimated using the kappa statistic. Data were reported as mean ± standard deviation (± SD) or median and interquartile range (IQR), as appropriate. Correlations between variables were assessed using Pearson’s rank correlation coefficient with a 95% confidence interval. The level of statistical significance was set at *p* < 0.05. Data were analysed using Matlab (The MathWorks, Natick, USA) and GraphPad (GraphPad Prism v10, California, USA).

## Results

### Clinical data

Thirty-seven patients were enrolled in the study; one was excluded because experts classified the corresponding CT scan as a focal injury. Of the 36 patients included, 31 received DECT. At least two experts classified the remaining thirty-six patients as diffuse injury, with initial disagreement for only one patient (*k* = 0.65, 95% CI from 0.02 to 1.00, indicating substantial agreement). The included thirty-six patients had a median age of 65 (IQR 56–68). The median time interval between ICU admission and CT scan was 4.5 days (IQR 1–9) and 3 days (IQR 1–8) from initiation of mechanical ventilation to CT scan. During the CT scan, the mean PEEP value was 13 cmH_2_0 (SD ± 3) and the ratio between partial pressure and an inspiratory fraction of oxygen (PaO_2_/F_I_O_2_) was 135 mmHg (IQR 105.0—171.8), confirming the ARDS diagnosis. Baseline demographic characteristics and comorbidities are shown in Table 1.

**Table 1:** Demographic data and respiratory parameters

Demographic data	All patients (*n* = 36)
Age^‡^(years)	65 (55–68)
Sex (male/total, %)	83
BMI	32 ± 7.8
Comorbidities	
Respiratory disease	7 (19%)
Hypertension	23 (63%)
Ischemic heart disease	3 (8.3%)
Heart failure	3 (8.3%)
Diabetes Mellitus	13 (36%)
Days on mechanical ventilation*^‡^	3 (1–8)
Days treated with neuromuscular blocking agent before CT-scan*^‡^	1 (1–3.8)
Respiratory parameters *	
Tidal volume (ml)	492.5 ± 106.4
Minute ventilation (ml/min)	11.5 ± 2.1
Peak pressure (cmH_2_O)	25.4 ± 5.7
PEEP (cmH_2_O)	13 ± 3.3
Dynamic compliance (ml/cm H_2_O) ^‡^	50.0 (36.1–68.2)
Tidal volume/PBW (ml/kg) ^‡^	6.9 (6.2–7.6)
PaO_2_/F_I_O_2_ (mmHg) ^‡^	135 (105.0–171.8)

Binary variables are expressed as absolute numbers and percentages (%); continuous variables are expressed as mean ± SD or median (IQR), denoted by ‡

Parameters collected at the point of the subject’s first CT examination. BMI, body mass index; PEEP, positive end-expiratory pressure; PBW, predicted body weight; PaO_2_/F_I_O_2_, partial pressure of arterial oxygen/fraction of inspired oxygen denoted by *

### CT image analysis

Hyperaeration ranged from 0 to 81% in the apical ventral areas, while non-aeration ranged between 1 and 84% in the dorsal diaphragmatic lung. The estimated lung weight was 1701 g (SD ± 818), whereas the total volume of gas within the lung was 1211 ml (SD ± 668).

### The focal index

**Fig. 3:**
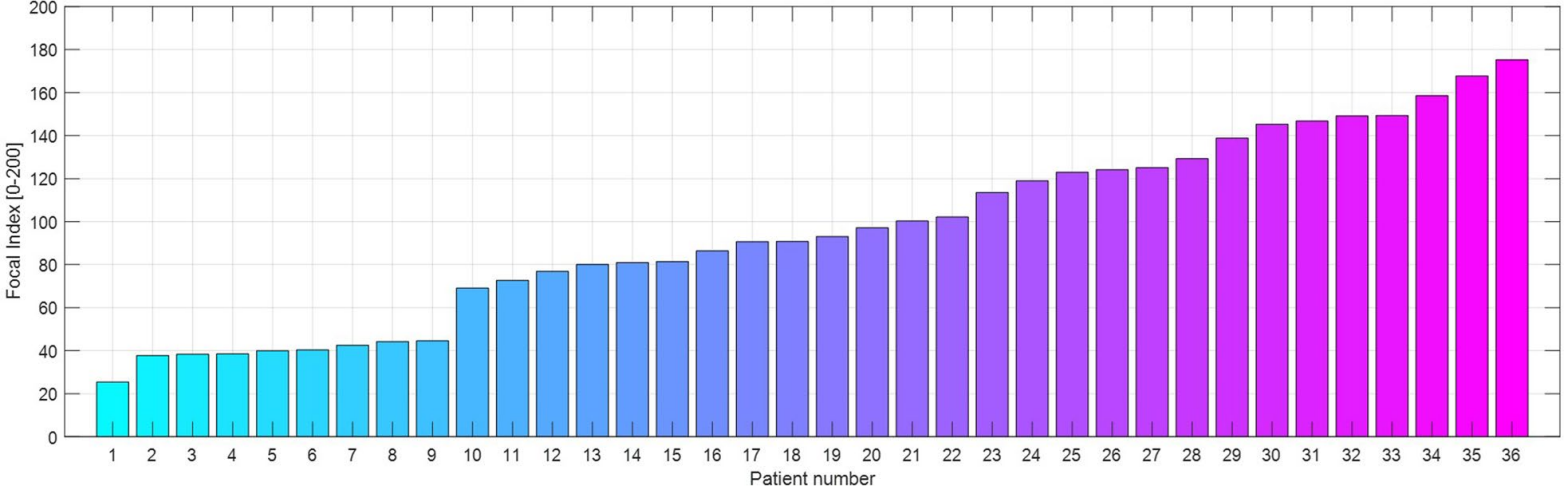
Focal index in the patients’ cohort. Histogram reporting the Focal CT score for the 36 patients included in the analysis. The focal index was calculated as the absolute difference between the areas under the curve for the HU distribution profile of the (Ventral Apical ROI) and the (Dorsal Diaphragmatic ROI)

**Fig. 4:**
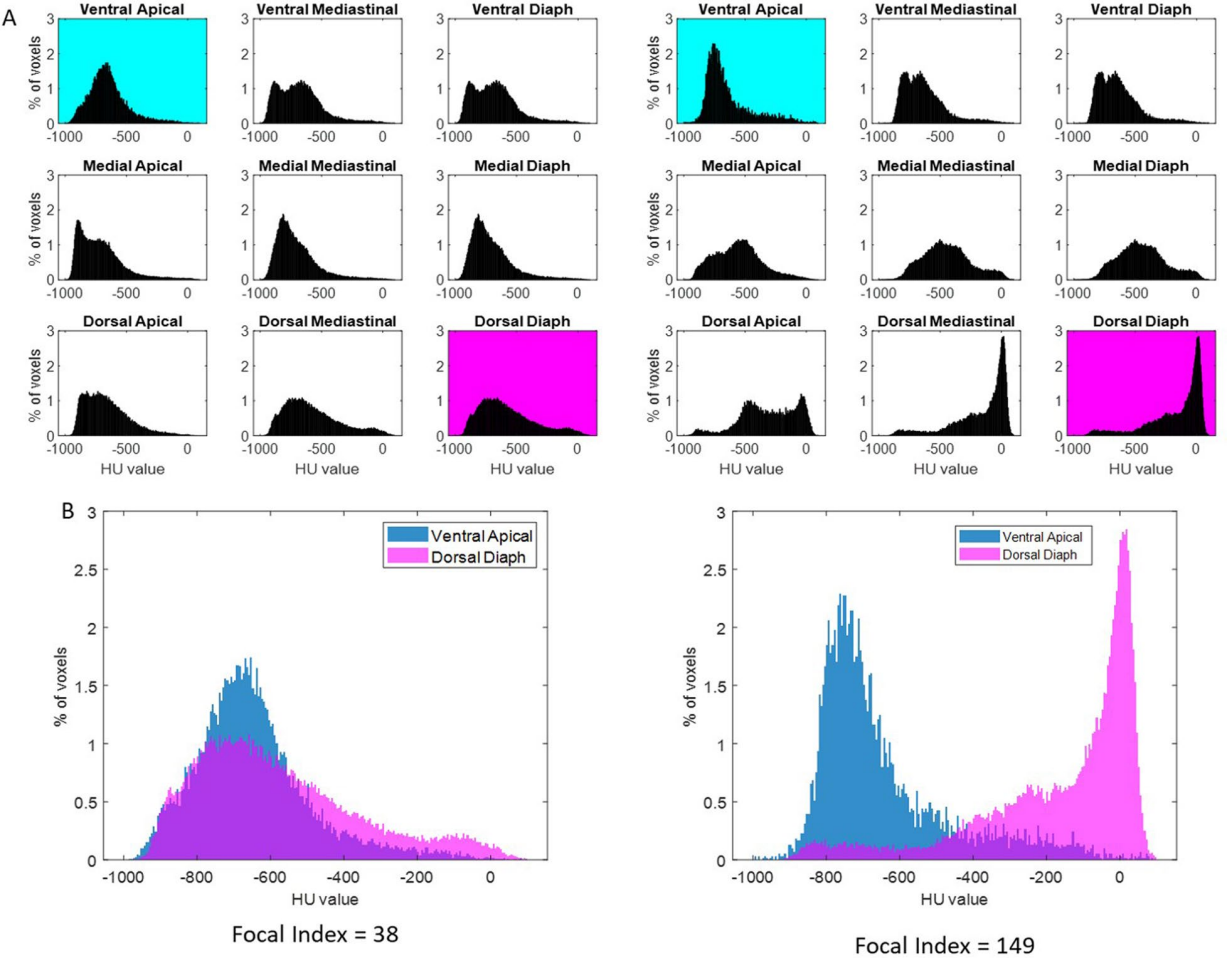
Regional HU distribution and focal index calculation in two representative patients. Two patients with two extreme values of the Focal index are reported. **A**. Regional HU distribution profiles for the nine defined three-dimensional regions of interest (ROI, i.e., Ventral Apical, Ventral Mediastinal, Ventral Diaphragmatic, Medial Apical, Medial Mediastinal, Medial Diaphragmatic, Dorsal Apical, Dorsal Mediastinal, Dorsal Diaphragmatic). The *Y*-axis reports the percentage of total voxels in that specific ROI, and the *X*-axis reports the HU values divided into 5 HU-wide bins. Histograms of HU distribution for each of the nine predefined lung regions in two patients: one with a low focal index (left, FI = 38) and one with a high focal index (right, FI = 149). The ventral apical (cyan) and dorsal diaphragmatic (magenta) regions are highlighted, as they are used for the focal index calculation. **B**. Superimposed HU distribution curves for the ventral apical and dorsal diaphragmatic regions. The focal index is calculated as the non-overlapping area between the HU distribution curves of the ventral apical and dorsal diaphragmatic regions of interest (ROIs). Each curve is normalised to an area of 100, and the focal index is computed as the integral of the absolute difference between the two curves across the full HU range (− 1000 to + 100). The resulting value is scaled by a factor of 100, yielding a score that ranges from 0 (complete overlap) to 200 (complete separation), reflecting the degree of morphological focality. The patient on the left shows substantial overlap between the two distributions, indicating low regional heterogeneity and a more uniform (diffuse) injury. In contrast, the patient on the right shows clear separation between the two regional HU profiles, reflecting pronounced dorsal consolidation and ventral preservation, consistent with a more focal pattern of injury despite expert classification as “diffuse”

**Fig. 5:**
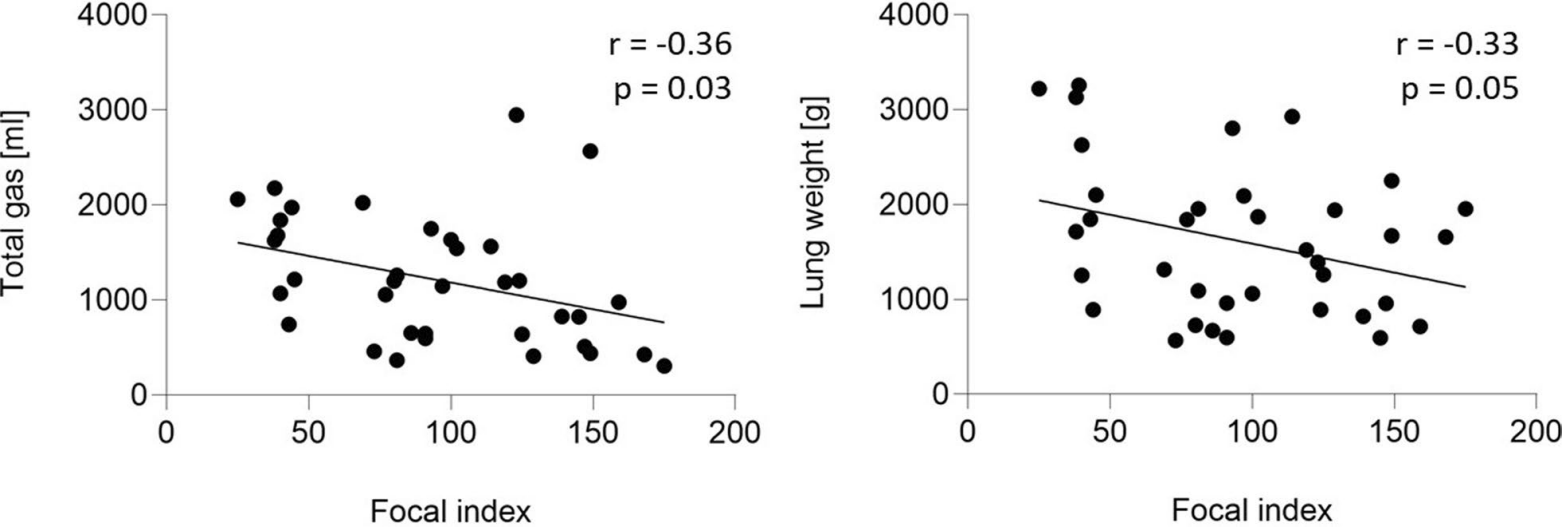
Pearson’s correlation to investigate the relationship between the focal index and: (1) total gas (left) and (2) lung weight (right)

The focal index ranged from 25 to 175 (Figs. 3 and 4), with a mean of 95.5 (SD ± 42.8). It significantly correlated with the dorsal diaphragmatic non-aerated area (*r* = 0.67, *p* < 0.01) but not with the ventral apical hyperaerated area (*r* = 0.13, *p* = 0.43). The focal index did not correlate with the cumulative fluid status of the patients at the moment of the CT scan (*r* = 0.17, *p* = 0.31). At the same time, it showed a significant indirect correlation with the total amount of gas within the lung parenchyma (*r* = − 0.36, *p* = 0.03). The correlation between the focal index and lung weight was indirect, but despite the apparent trend, it did not reach statistical significance (*r* = − 0.33, *p* = 0.05) (Fig. 5).

### Sensitivity analysis

**Fig. 6:**
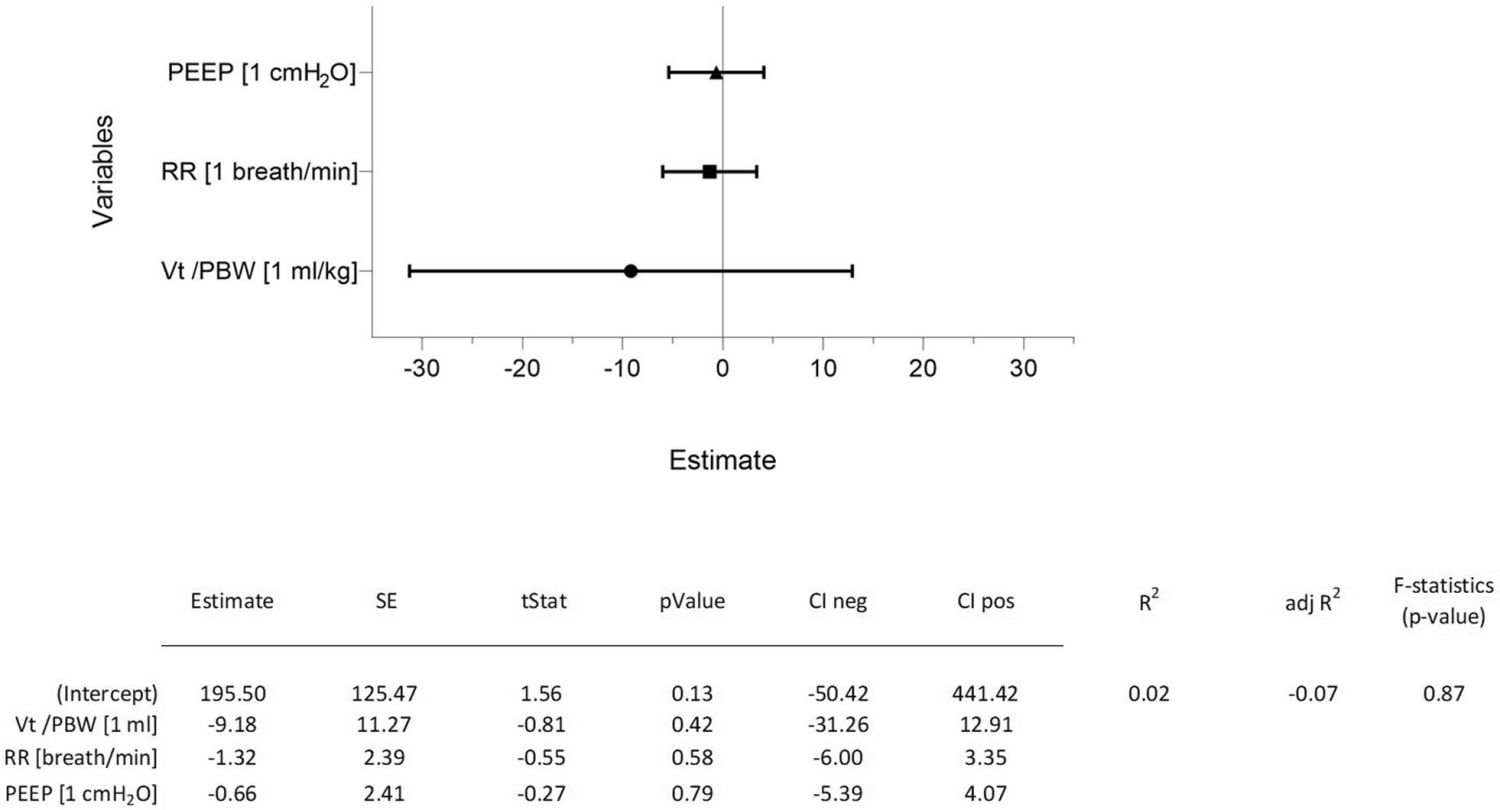
Sensitivity analysis testing the influence of ventilatory variables on the focal index. The three independent variables tested were (1) tidal volume per predicted body weight (Vt/PBW, ml/kg), (2) respiratory rate (RR, breaths/minute), and (3) positive end-expiratory pressure (PEEP, cmH_2_O). The multiple linear regression model tested their effect on the focal index, showing the absence of any significant influence

The analysis did not show a significant influence of any tested ventilation-related variables on the focal index (*R*^2^ value = 0.02; adj *R*^2^ = − 0.07 *F*-statistic, *p*-value = 0.87) (Fig. 6).

## Discussion

Lung morphology has the solid potential to be a discriminating factor for individualising ventilatory strategies in ARDS patients [8]. However, previous attempts to use lung morphology to individualise ventilatory strategy are based on operator-dependent evaluations [10, 14]. In the current analysis, for the first time, we clearly showed an overlay of what is defined as diffuse or focal injury in the morphological evaluation of ARDS, challenging the current binary classification of focal versus diffuse injury. Focal and diffuse ARDS may exist along a continuous spectrum of focality in lung injury. This entails a constant inherent risk of misclassifying lung injury as diffuse or focal and, consequently, applying suboptimal ventilatory strategies based on inaccurate morphological categorisation. A recent multicentre randomised trial misclassified as much as 21% of the CT scans or chest *X*-rays analysed [10], leading to a significant increase in mortality for those subjects enrolled in the incorrect intervention group. This issue applies not only to CT, which is considered the gold standard for the morphological classification of ARDS patients, but also to other types of lung imaging. Therefore, an objective morphological index is required to have a univocal sub-phenotype classification. Our choice of dividing the lung into nine regions along both the gravitational axis (from ventral to dorsal) and the craniocaudal axis (from the apex to the diaphragmatic regions) is derived from the known gradient of transpulmonary pressure along both axes [21, 22]. Most of the dorsal and caudal regions of the lungs are subjected to significantly lower end-expiratory transpulmonary pressures than most ventral and apical regions. Already valid for physiological conditions, this is accentuated in ARDS patients, where several factors contribute to further magnifying this gradient. Among others, intra-abdominal pressure, lung collapse and sedation cause a cranial displacement of the diaphragmatic dome and a redistribution of pressures.

While a significant increase in ventral gas content has been previously shown in CT scans classified as focal [20], we found a significant correlation between the focal index and the dorsal diaphragmatic non-aerated lung, but not between the focal index and the ventral apical hyperaerated lung. This highlighted the prevalence of dorsal peri-diaphragmatic lung collapse over the onset of ventral apical hyperaeration in the transition between diffuse and focal injury.

The current study also investigated whether the patient’s fluid status and lung weight, a surrogate of lung oedema estimated from CT analysis, influenced lung morphology and could be detected by the focal index. While the overall patient’s fluid status did not correlate with the degree of focality of the lung injury, a trend towards a lower lung weight while increasing focal injury was found in our patient cohorts. This finding aligns with previous literature showing that ARDS is characterised by injury of the alveolar-capillary membrane, leading to high permeability and impaired clearance of alveolar fluids, mainly occurring in diffuse rather than focal injury [23]. Indeed, previous studies have demonstrated an association between diffuse damage, lung oedema, and the inflammatory state of the lung [24, 25] that is not necessarily linked to the overall fluid status of the patient. The degree of focality of lung injury was also inversely correlated to the total amount of lung air, resulting from the predominant loss of aeration in the collapsed non-aerated regions, characterising focal injury [20].

Of clinical relevance was the finding that setting ventilatory parameters within the limits used during CT image acquisition did not influence the lung’s morphological appearance and focal index. This suggested that rather than ventilatory settings, intrinsic patient factors and injury patterns primarily dictate the morphological lung sub-phenotype. This independence from ventilator parameters enhances the utility of the focal index as an objective metric for classifying lung injury. Although this was true for the ranges of values selected in the studied cohort, it is not necessarily valid for all values of PEEP, tidal volume, and respiratory rate. A previous study showed the divergent effects of 10 cmH_2_O of PEEP, when compared to 0 cmH_2_O, on focal and diffuse ARDS [14], which may impact the computation of the focal index.

The present pilot study was designed to introduce a quantitative descriptor of regional heterogeneity rather than to prescribe ventilator settings. Nonetheless, once validated in a broader ARDS population, the focal index could support graded, rather than strictly dichotomous, adjustments of mechanical ventilation strategies. Current morphology-based protocols [10, 13] typically allocate patients with “focal” injury to lower PEEP, liberal tidal volume (8 ml/kg PBW), and early prone positioning. In contrast, those with “diffuse” injury receive higher PEEP and more restrictive tidal volumes. A continuous index offers the possibility of tailoring therapy along a spectrum: patients with very low focal-index values (minimal regional disparity) might benefit from recruitment manoeuvres and higher PEEP, patients with very high values (marked dorsal consolidation with ventral sparing) may respond preferentially to prone positioning and conservative PEEP. In contrast, those with intermediate values could be managed with intermediate levels of PEEP and tidal volume. These hypotheses remain speculative and can be tested prospectively only after external validation of the index and definition of clinically relevant cut-offs.

This preliminary analysis highlights the common coexistence of focal and diffuse lung injury, emphasising the need for an objective score to quantify and characterise lung injury and enable an objective classification into different subphenotypes. In a clinical context, the focal index can be a valuable tool for bedside decision-making in ventilator settings or rescue manoeuvres that suit the lung at that given time. From these preliminary results, a low index suggests a more diffused lung injury, possibly indicating the patient could benefit from ventilatory recruitment manoeuvres and higher levels of PEEP. In contrast, a higher index may require a more precise evaluation of the focality and may be more responsive to recruitment with positional changes, such as prone positioning. However, this preliminary analysis entailed some limitations. First, it included only one etiological sub-phenotype of ARDS (i.e., COVID-19 ARDS). This was an intended choice to select a homogeneous population of patients with diffuse lung injury. Investigating a broader cohort with diverse ARDS aetiologies, including both diffuse and focal morphologies, is warranted to obtain a more general validation of the focal index. Second, although the focal index is continuous by design, future work should aim to define clinically relevant thresholds (e.g., a cut-off value indicative of focal, diffuse or intermediate injury) to support bedside applicability. Third, a further potential limitation is that CT scans were not standardised to specific phases of the respiratory cycle (e.g., end-expiration), which could partially influence regional aeration and thus the focal index.

Building on this preliminary analysis, we are currently conducting a follow-up study that includes a more diverse ARDS population, encompassing multiple etiologies and both focal and diffuse presentations. This broader cohort will enable a direct comparison between expert classifications and the focal index, allowing for a more comprehensive validation of the index’s diagnostic potential. In a broader, more heterogeneous cohort, future studies will explore how the focal index correlates with clinical and patient-centred outcomes, such as survival, ventilator-free days, and response to prone positioning, to assess its potential prognostic and therapeutic value. Given the limited heterogeneity in expert classification (only one case rated as focal), the estimation of interobserver agreement was inherently imprecise, with a wide CI (0.02–1) for the kappa statistic. This underscores the need for validation in a more morphologically diverse ARDS population.

## Conclusions

Accurate classification is needed to personalise ventilation strategies based on lung morphology. This study highlights in patients with COVID-19 and ARDS the limitations of current subjective classifications of ARDS morphology, demonstrating that diffuse lung injury encompasses a spectrum of focality. The Focal CT index provides a quantifiable approach to evaluating regional variations in lung injury, offering a potential tool for guiding personalised ventilation.

## Supplementary Information

Additional file 1.

## Data Availability

Data are available from the corresponding author upon reasonable request.

## Abbreviations

ARDS: Acute respiratory distress syndrome
BMI: Body mass index
CT: Computed tomography
F_I_O_2_: Inspiratory fraction of oxygen
HU: Hounsfield unit
ICU: Intensive care unit
IQR: Interquartile range
LUS: Lung ultrasound
PaO_2_: Partial pressure of oxygen
PBW: Predicted body weight
PEEP: Positive end-expiratory pressure
RR: Respiratory rate
SD: Standard deviation
Vt: Tidal volume
