# Supplementary figures and images for: The focal index: a quantitative approach to morphological sub-phenotyping of COVID-19 patients with acute respiratory distress syndrome: a pilot study
Source: Intensive Care Med Exp. 2025 Aug 8;13:81. doi: 10.1186/s40635-025-00794-0 (PMC12334779; doi:10.1186/s40635-025-00794-0)

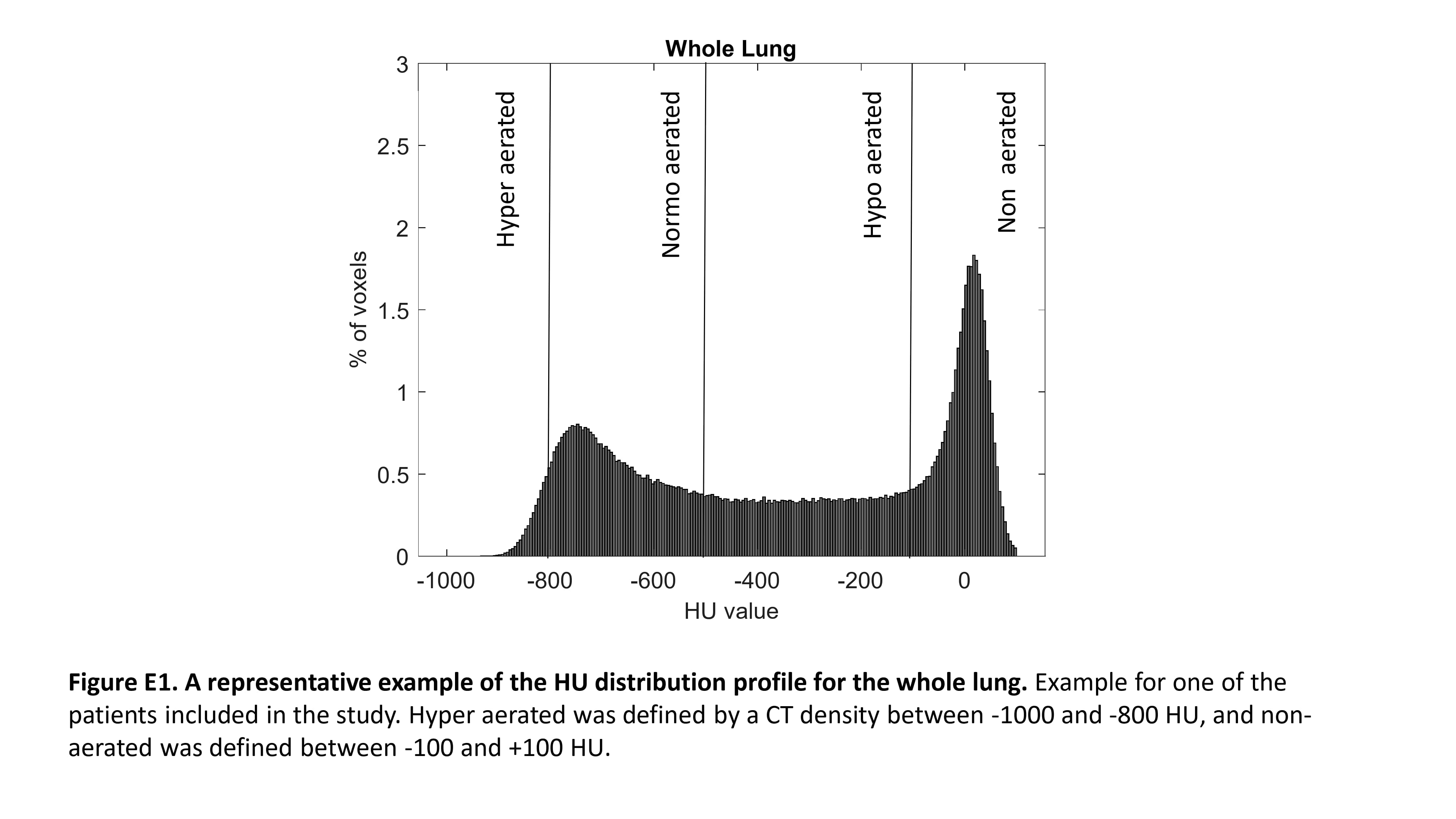

Supplement: Supplementary file 1 — Additional file 1. [file 40635_2025_794_MOESM1_ESM.jpg]
